# Successful emergent repair of a subacute left ventricular free wall rupture after acute inferoposterolateral myocardial infarction

**DOI:** 10.1186/s13019-018-0764-z

**Published:** 2018-06-28

**Authors:** Arjan J. F. P. Verhaegh, Wobbe Bouma, Kevin Damman, M. Nasser Morei, Massimo A. Mariani, Joost M. Hartman

**Affiliations:** 1 Department of Cardiothoracic Surgery, University of Groningen, University Medical Center Groningen, P.O. Box 30001, 9700 RB Groningen, The Netherlands; 2 Department of Cardiology, University of Groningen, University Medical Center Groningen, P.O. Box 30001, 9700 RB Groningen, The Netherlands; 3 Department of Anesthesiology and Pain Medicine, University of Groningen, University Medical Center Groningen, P.O. Box 30001, 9700 RB Groningen, The Netherlands

**Keywords:** Myocardial infarction, heart rupture, percutaneous coronary intervention, cardiac surgical procedures

## Abstract

**Background:**

Myocardial rupture is an important and catastrophic complication of acute myocardial infarction. A dramatic form of this complication is a left ventricular free wall rupture (LVFWR).

**Case presentation:**

A 70-year-old man with acute inferoposterolateral myocardial infarction and single-vessel coronary artery disease underwent emergency percutaneous coronary intervention (PCI). The circumflex coronary artery was successfully stented with a drug-eluting stent. Fifty days after PCI the patient experienced progressive fatigue and chest pain with haemodynamic instability. Transthoracic echocardiography showed a covered LVFWR of the lateral wall. The patient underwent successful emergent surgical repair of the LVFWR.

**Conclusions:**

In the current era of swift PCI, mechanical complications of acute myocardial infarction, such as LVFWR, are rare. The consequences, however, are haemodynamic deterioration and imminent death. This rare diagnosis should always be considered when new cardiovascular symptoms or haemodynamic instability develop after myocardial infarction, even beyond one month after the initial event. Timely diagnosis and emergency surgery are required for successful treatment of this devastating complication.

## Background

In patients with acute myocardial infarction (AMI), myocardial rupture is an important and catastrophic complication, which directly causes death in approximately 8% of the patients [1]. An infrequent but dramatic form of this complication is a left ventricular free wall rupture (LVFWR). Herein, we report the presentation and successful treatment of a septuagenarian, who presented with a subacute left ventricular free wall rupture (LVFWR) almost two months after swift emergency percutaneous coronary intervention (PCI) for acute inferoposterolateral myocardial infarction.

## Case presentation

A 70-year-old man with a history of hypertension, insulin-dependent diabetes mellitus, and a carotid endarterectomy was admitted to our emergency department with persisting and progressive angina for two hours. Moreover, he complained about general malaise and decreased exercise tolerance in the week prior to hospital admission. On physical examination, cardiac sounds were normal on auscultation. His blood pressure was 110/60 mm Hg and heart rate was 90 bpm. On admission, serum troponin T was 956 ng/L (normal range [NR] < 14 ng/L), creatine kinase myocardial band (CK-MB) 18 U/L (NR < 25 ng/L), creatine kinase (CK) 132 U/L (NR < 132 ng/L), low-density lipoprotein (LDL) cholesterol 3.4 mmol/L, high-density lipoprotein (HDL) cholesterol 0.8 mmol/L, triglyceride 0.75 mmol/L, and C reactive protein 150 mg/L (NR < 5 mg/L). His electrocardiogram (ECG) showed sinustachycardia (118/min) with ST-segment elevation in leads II, III, aVF and V5–6 and ST-segment depression in leads V1-V2. Coronary angiography was performed within one hour of hospital admission and revealed single-vessel coronary artery disease and the patient underwent a percutaneous coronary intervention of the circumflex coronary with placement of a drug-eluting stent. After the procedure, a Thrombolysis In Myocardial Infarction (TIMI) grade 2 flow was achieved in the AMI culprit coronary artery. Peri-procedural hypotension was treated with infusion of 2 l of 0.9% sodium chloride and dobutamine infusion. Postprocedural transthoracic echocardiography showed a moderate left ventricular function with a left ventricular ejection fraction of 40–45% and trivial mitral and tricuspid regurgitation.

**Fig. 1:**
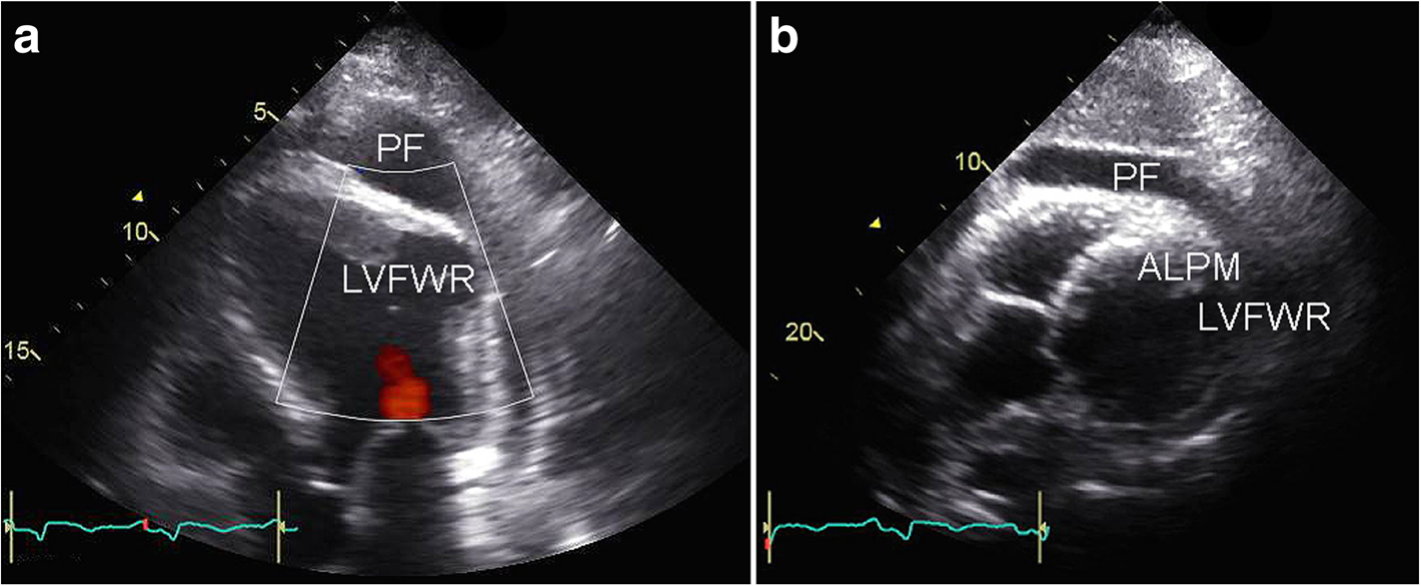
Preoperative transthoracic echocardiographic imaging. Preoperative transthoracic echocardiography showing covered left ventricular free wall rupture (LVFWR) of the lateral wall in the apical four-chamber view (**a**) and the parasternal short-axis view (**b**). Note extensive pericardial fluid (PF) and the close proximity of the rupture to the anterolateral papillary muscle (ALPM)

Fifty days after PCI the patient experienced progressive fatigue and chest pain with haemodynamic instability. Transthoracic echocardiography showed a covered left ventricular free wall rupture (LVFWR) of the lateral wall and extensive pericardial fluid and inflow obstruction. The free wall rupture was in close proximity to the anterolateral papillary muscle (ALPM), but did not lead to significant mitral regurgitation. The transthoracic echocardiographic images are shown in Fig. 1.

**Fig. 2:**
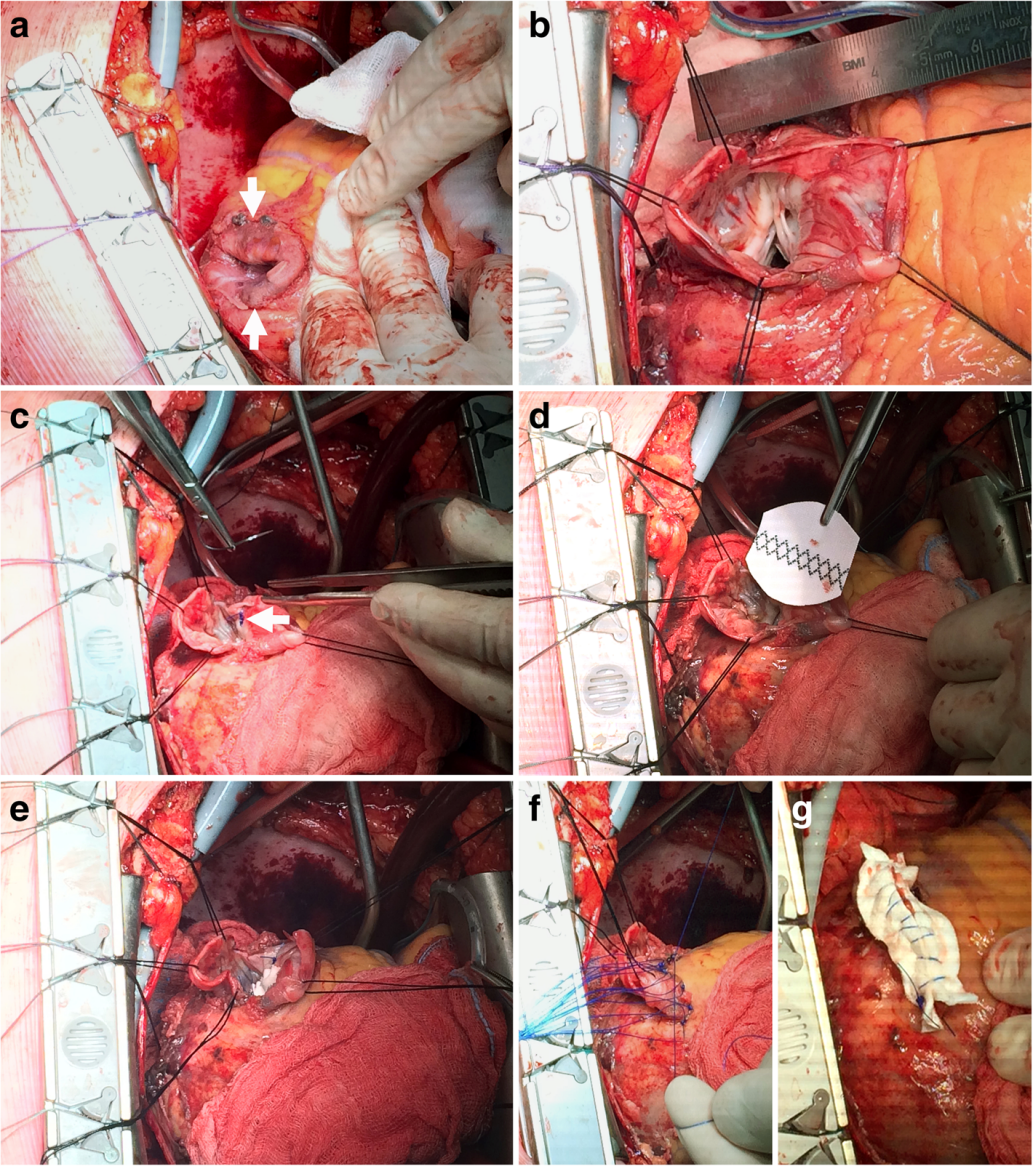
Intraoperative photographs. After carefully opening the chest and pericardium, cardiopulmonary bypass was instituted. Subsequently, cardioplegic cardiac arrest was achieved and the covered left ventricular free wall rupture of the lateral wall was visualized after careful manupulation of the heart (**a**, arrows). The left ventricular free wall rupture was only covered by a thin layer of epicardium. Opening of the thin-walled epicardial layer revealed the close proximity of the rupture to the anterolateral papillary muscle of the mitral valve (**b**). The defect was approximately 5–6 cm in diameter. The LVFWR was repaired by (1) approximation of the defect with a circumferential purse-string suture (a so-called “Fontan stitch”) (**c**, arrow), which reduced the diameter of the defect to 3 cm, (2) securing a Dacron patch on the endocardial surface of the heart (carefully avoiding sutures too close to the ALPM) (**d**, **e**) and (3) subsequently closing the ventriculotomy in two rows (a deep row with horizontal interrupted mattress sutures (**f**) and a superficial row with a continuous suture over a double layer of felt (**g**))

The patient was brought to the operation room for emergent repair of the rupture. The surgical technique is described and shown in Fig. 2. The patient was weaned of cardiopulmonary bypass through the use of mild doses of inotropes. Transesophageal echocardiography showed a good result without significant mitral regurgitation. By carefully avoiding damage to the ALPM during the procedure we were able to avoid additional mitral valve repair or replacement.

Postoperative recovery was uneventful and the patient was discharged 12 days after the operation. More than a year later, the patient did not experience any subsequent cardiac events and his functional status was similar to the level before the episode of myocardial infarction and rupture.

## Discussion and conclusions

In the current era of swift percutaneous coronary intervention, left ventricular free wall ruptures (LVFWR) have been reported in < 1% of patients with acute transmural myocardial infarctions [2, 3]. Although rare, LVFWR occurs up to ten times more frequently than other fearsome complications of acute myocardial infarction, such as intraventricular septum and papillary muscle ruptures, and is associated with a high mortality accounting for approximately 20–30% of all infarct related deaths [2, 4]. Traditionally, risk factors for LVFWR include advanced age (6^th^ decade or later), female gender, hypertension without left ventricular hypertrophy, delayed or no reperfusion, anterior location of the AMI, first (usually transmural) myocardial infarction, no history of angina, poor collateral circulation and use of NSAIDs or corticosteroids during the acute phase [2–7]. Although these risk factors are renowned, they are not specific enough to predict which patients are at risk of rupture [8].

LVFWR occurs generally between one and seven days after myocardial infarction, but rupture has been reported to occur as late as one month or even beyond as illustrated by our case [5, 6, 9, 10]. According to the type of presentation, LVFWR can be divided in an acute and subacute form [11]. The acute form of LVFWR is associated with a prompt haemodynamic collapse due to severe hypotension and electromechanical dissociation secondary to acute cardiac tamponade. Death ensues usually in a matter of minutes to hours, as resuscitative manoeuvres are uniformly unsuccessful [11–14]. The less frequent, subacute form evolves more slowly and gradually over hours to even days. In these cases, the rupture is often sealed by epicardium or by a haematoma on the epicardial surface of the heart, forming a contained and covered myocardial rupture. In pathological terms, this condition stands somewhere between the insidious free rupture into the pericardial cavity and the gradual formation of a pseudoaneurysm [6, 15–17]. This subacute or “oozing type” rupture presents mainly with pericardial effusion related signs and symptoms.

LVFWR should be suspected in patients with recent myocardial infarction who experience recurrent or persistent (severe) chest pain, intractable vomiting, haemodynamic instability, arrhythmias, syncope (due to transient electromechanical dissociation) and signs of cardiac tamponade [6, 14, 17]. Electrocardiographic findings in LVFWR may be related to its type and severity. New or persistent (characteristically so-called ‘saddle-shaped’) ST-segment elevations and persistent positive T-wave deflections may indicate the subacute variety, while electromechanical dissociation and bradycardia are features of the acute form [9, 10, 13].

When a patient is suspected of having a LVFWR, the preferred diagnostic modality to confirm the diagnosis is swift bedside echocardiography (transthoracic or transesophageal) [3, 10, 12, 17]. The diagnostic accuracy of echocardiography, in terms of sensitivity and specificity, in the diagnosis of LVFWR approaches 100% [9, 12, 17]. The most common echocardiographic finding in patients with LVFWR are pericardial effusion. Occasionally, the actual myocardial tear or rupture site can be visualized [9, 10, 17]. Although pericardial effusion may result from a broad spectrum of etiologies, LVFWR should always be considered the leading diagnosis in patients with a recent AMI and haemodynamic instability.

Despite cases with fairly good survival rates following conservative approaches have been reported [18], it is general accepted that emergent surgical repair is the cornerstone treatment for patients with (subacute) LVFWR as it provides the only potentially definitive treatment option [12]. Several surgical techniques have been described. All involving extensive debridement (infarctectomy) into normal cardiac tissue followed by direct linear closure or endoventricular patch plasty, while preserving left ventricular geometry [3, 19, 20]. Lately, off-pump sutureless techniques using biocompatible hemostatic glues and patches to cover the necrotic and ruptured area are increasingly explored with rather good results [21–23]. Coronary artery bypass grafting is advocated as needed, because myocardial rupture is associated with multivessel coronary artery disease in the majority of patients [7]. Therefore, it may be considered to perform emergency coronary angiography in the subacute setting to determine which coronary arteries to bypass. If the patient’s clinical condition does not permit this delay, the possibility of directly proceeding to surgery and performing empirically based bypassing of all major coronary arteries has been described [7, 12]. The emerging use of hybrid operating rooms, enabling intraoperative coronary angiography, may circumvent the latter problem. Since our patient had recently undergone coronary angiography, we were not faced with this dilemma.

Despite high perioperative mortality rates (up to 50%), long-term survival with rather well preserved left ventricular and good functional outcome has been accomplished, as also shown in our case [7, 9, 10, 17]. This may become more common, when timely diagnosis of this devastating complication will be improved.

As illustrated in this case, LVFWR can occur even beyond one month after myocardial infarction followed by swift PCI and is – unless recognized immediately – not always fatal, especially in patients with the subacute form of presentation. Improved identification of patients at risk, better acquaintance of the clinic presentation and expeditious application of bedside echocardiography are warranted for timely diagnosis allowing enough time for surgical salvage.

## Abbreviations

ALPM: Anterolateral papillary muscle
AMI: Acute myocardial infarction
LVFWR: Left ventricular free wall rupture
PCI: Percutaneous coronary intervention
PF: Pericardial fluid
TIMI: Thrombolysis in myocardial infarction
